# Disrupted functional connectivity between the periaqueductal gray and other brain regions in a rat model of recurrent headache

**DOI:** 10.1038/s41598-017-04060-6

**Published:** 2017-06-21

**Authors:** Zhihua Jia, Wenjing Tang, Dengfa Zhao, Shengyuan Yu

**Affiliations:** Department of Neurology, Chinese PLA General Hospital, Beijing, 100853 China

## Abstract

Functional connectivity (FC) has been used to investigate the pathophysiology of migraine. We aimed to identify atypical FC between the periaqueductal gray (PAG) and other brain areas in rats induced by repeated meningeal nociception. The rat model was established by infusing an inflammatory soup (IS) through supradural catheters in conscious rats. Quiescent and face-grooming behaviors were observed to assess nociceptive behavior. FC analysis seeded on the PAG was performed on rats 21 days after IS infusion. The rats exhibited nociceptive behavior correlates of human behaviors associated with migraine after IS infusion. The PAG showed increased FC with the prefrontal cortex, cingulate gyrus, and motor cortex but decreased FC with the basal ganglia, dorsal lateral thalamus, internal capsule and prelimbic cortex in the rat model. The atypical FC of the PAG with brain regions in the rat model that are involved in nociception, somatosensory processing, emotional processing, and pain modulation are consistent with the clinical data from migraineurs, indicate that resting-state FC changes in migraine patients may be a consequence of headache attacks, and further validate this rat model of chronic migraine.

## Introduction

Primary headache is one of the most common disorders of the nervous system, with a 1-year prevalence rate of 47% among the global population^1^. Migraine is the most common primary headache disorder that is presented in physicians’ offices^2^, and is more common in women (17.6%) than men (6.5%)^3^. Migraine has high socio-economic and personal effects and was ranked as the seventh leading cause of disability worldwide in the Global Burden of Disease Survey, 2015^4^. One subtype of migraine, chronic migraine, is defined by headaches occurring on >15 days per month for longer than 3 months, which has the features of migraine headache on at least 8 days per month^5^. This subtype has a global prevalence of ~2%, specifically, 1.7–4.0% in women and 0.6–0.7% in men^6^. Nevertheless, the pathophysiology of chronic migraine remains unclear. This lack of clarity makes disease monitoring, treatment, and prevention difficult. A prevailing theory of the pathogenesis of migraine attacks is that hyperexcitability develops along the trigeminovascular pathway^7^ and is probably facilitated by dysfunction in the descending pain modulatory circuits^8–10^.

The periaqueductal gray (PAG) is a key region that is involved in both the trigeminovascular pathway of pain^7^ and endogenous pain inhibition^11^. The relevance of the PAG to the pathogenesis of migraine was first suggested in reports of subjects without headaches who developed migraine-like episodes after stereotactic placement of electrodes in this area of the brainstem^12^. Moreover, the presence of a midbrain plaque in multiple sclerosis (MS) patients was reported to be associated with an increased likelihood of headaches with migraine characteristics^13^. Moreover, PAG activation has been observed during migraine attacks that occurred spontaneously in a positron emission computed tomography study^14^. Involvement of the PAG in migraine has also been demonstrated through iron homeostasis impairment^15^, increased gray matter density^16^ and significant increases in mean kurtosis (MK) and mean diffusivity (MD) values in magnetic resonance imaging (MRI) studies^17^. Preclinical research has revealed that afferent trigeminal nociceptive traffic is inhibited by stimulation of the PAG^18^, and molecules related to inflammation and pain, such as calcitonin gene-related peptide (CGRP), act in the PAG^19^. All of these findings indicate that the PAG plays an important role in the pathogenesis of migraine.

Because migraine is mainly a disorder of brain function, functional MRI (fMRI) studies are useful for studying the underlying mechanisms of migraine. Functional connectivity (FC) is a descriptive measure of spatiotemporal correlations between distinct cerebral regions^20^. Resting-state functional connectivity (rs-FC) has been used to investigate the pathophysiology of neuropsychiatric disorders, such as Alzheimer’s disease^21^, schizophrenia^22^, and cluster headaches^23^. PAG seed-based rs-FC studies have been performed in interictal migraineurs^24–27^ and have suggested that rs-FC may be useful in revealing the pathophysiology of migraine. Despite the important information obtained from imaging studies of migraineurs, no FC study has been reported in animal models of migraine for basic research.

The commonly used animal model of chronic migraine involves repeated infusion of inflammatory soup (IS) through a transcranial cannula to stimulate trigeminovascular and meningeal afferents. The validity and reliability of this model of chronic migraine has been proven by mimicking the chronic migraine phenotype (i.e., behavioral quiescence, intense hemifacial touching during attacks and cutaneous allodynia)^28, 29^, an increase in extracellular glutamate in the trigeminal nucleus caudalis (TNC)^30^, increased expression of CGRP in the medulla^31^ and a response to triptans^29^. However, the functional brain changes in this animal model of chronic migraine are unknown.

Thus, in the present study, we used fMRI to investigate FC within the PAG in rats exposed to repeated dural inflammatory stimulation. We chose the animal model described by Wieseler, because the polyurethane tubing used is suitable for MRI scanning^28^. We sought to test the hypothesis that there is atypical FC of the PAG with brain areas related to nociception, emotion processing, and pain modulation which may be consistent with clinical studies in migraineurs. If there are changes concordant with those in rs-FC studies of migraineurs, such findings may further validate the applicability of this as a rat model of chronic migraine and could provide a new approach for future research using MRI to study migraine.

## Materials and Methods

Twelve specific-pathogen-free Sprague Dawley male rats (weight, 180–220 g; age, 6–7 weeks) were used. Because pain was induced, the number of rats studied was restricted to the minimum necessary to run statistical analyses (*n* = *6*/group). The rats were housed individually in a temperature-controlled (22 ± 2 °C) environment under a 12/12-h light/dark cycle and allowed food and water ad libitum.

The experimental procedures were approved by the Laboratory Animal Center of the General Hospital of the Chinese People’s Liberation Army (Beijing, PR China) and were consistent with the ethical guidelines recommended by the International Association for the Study of Pain in experimental and conscious animals^32^.

### Surgical procedures

The PE10 tubing preparation was conducted according to the methods of Wieseler *et al*.^28^ and modified by bending a 1 mm section from the end of the catheter to 90°. This was then be oriented under the skull during surgery to avoid dural damage.

Following an habituation period, the rats were placed under general anesthesia (pentobarbital 50 mg/kg, intraperitoneal) and positioned in a stereotaxic apparatus (ZS-B/C, Beijing, China). All surgical tools were sterilized. For local anesthesia, 0.5 mL of lidocaine hydrochloride (0.1 g/5 mL) was applied subcutaneously to the skull in the region of the craniotomy. Next, a craniotomy was performed as described in Wieseler *et al*.^28^. Two 8–10-mm long, 2-mm wide and ~0.5-mm deep troughs were drilled in the skull to orient and secure the PE10 tubing, and then the bent ends of the catheters were oriented into the drilled holes and placed onto the dural surface; the other parts of catheters were laid horizontally along the troughs. The catheters were flushed with 5 μL of sterile saline through a 10 μL syringe. The catheters were then attached to the skull using 502 glue (an ethyl-cyanoacrylate-based glue) and dental cement (a methyl methacrylate resin-based type). The incised skin was then sutured. The rats recovered in a heated recovery box and were then individually housed. After surgery, the rats recovered for approximately 1 week before use in the experiments.

### Experimental procedures

**Figure 1:**
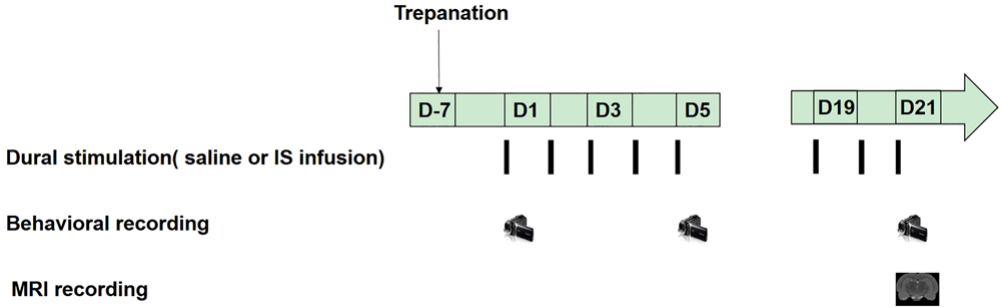
Schematic of the experimental design. About 1 week after surgery, the rats received daily inflammatory soup (IS) or saline infusion. Nociceptive behaviors were recorded once every 4 days in both groups before and after infusion. Magnetic resonance imaging (MRI) recordings were performed on day 21.

IS (2 mM histamine, 2 mM 5-HT, 2 mM bradykinin, and 0.2 mM PGE2 in sterile saline) was made from stock solutions just prior to use^28^. The animals were divided randomly into two equally sized groups according to a sequence generated by a random-number table to avoid bias in selection, i.e., the IS group (*n* = *6*) and Control group (*n* = *6*). The rats in the IS group received infusions of IS (10 μL) for 5 min daily for 21 days^33^, and the Control group received sterile saline. The rats were infused with IS or saline daily for 21 days to approximate the headache frequency of patients with chronic daily headache; the International Headache Society classifies chronic migraine as that occurring >15 days per month^5^. All cannula placements on top of the dura were verified on MRI scans and post mortem. The experimental design is illustrated in Fig. 1.

The experiments were conducted between 8:00 and 14:00 during the daylight portion of their circadian cycle. The rats were placed in a plastic tube restraint for IS/saline infusion. The behavioral recordings were performed according to the methods of Dong *et al*.^34^. The rats were recorded for 15 min before the infusion and for another 30 min after the infusion. The same procedure was repeated once every 4 days. The quiescent and face-grooming behaviors were observed. Quiescent behavior includes resting and freezing behavior, and the time spent in quiescent behavior was analysed using the EthoVision XT animal tracking software (ver. 9.0), which is a program that is used to automatically analyse the behavior, movement, and activity of animals in an open-field environment (Noldus Information Technology, Wageningen, the Netherlands)^35^. Face-grooming was characterized by rubbing the face or scratching the head with the limbs and the total time spent displaying face-grooming behavior was measured manually (in s), in a blinded manner with a chronometer for periods of 3 minutes^29^. All data are presented as the means ± the standard deviations (SDs).

### fMRI acquisition and FC analysis

At 3 weeks after the daily infusion of IS or saline, the MRI data were acquired using a 7.0-T Bruker Pharma Scan system (Bruker BioSpin, Ettlingen, Germany) with a 38-mm-diameter birdcage coil. For the MRI data collection, the rats were anesthetized with isoflurane (5% for initial induction and 1.5% during MRI scanning) in a gas mixture of 40% O2 and 60% N2^36^. Each rat was placed in the prone position on an MR-compatible stereotactic holder with a bite bar and a gas mask to exhaust the isoflurane in a mixture of oxygen and air. The body of each rat was fixed to the holder with tape. Respiration rate was monitored using a pressure sensor (SA Instruments, Stony Brook, NY, USA) throughout the scans and maintained at a rate of 40–50 breaths per min by controlling the level of isoflurane/oxygen mixture. Cephalic mechanical allodynia was prominent in the rats at 1 hour after IS infusion and returned to preinfusion values within 4–5 hours after repeated IS infusion^9^; thus, the rats were imaged 24 h after the last infusion of IS to mimic an interictal migraine state.

High-resolution anatomical MRI data were collected using a T2-weighed RARE sequence. These T2-weighted images (T2WI) were obtained using a 2D-RARE sequence with the following parameters: TR = 6,200 ms, TEeff = 24 ms, flip angle = 180°, FOV = 35 × 35 mm^2^, matrix size = 256 × 256, slice thickness = 0.3 mm, slice gap = 0 mm, and total scan time = 20 min^37^. Functional images were obtained using a gradient echo-planar imaging (EPI) sequence (TR = 2,000 ms, TE = 27.1 ms, flip angle = 90, slice thickness = 1 mm, slice gap = 0 mm, matrix = 128 × 128), and 150 continuous EPI functional volumes were acquired axially over 13 min 20 s^38^.

All functional image post-processing was performed by a single, experienced observer who was blinded to the treatment group. The preprocessing and data analysis were performed using the “spmratIHEP” toolbox^39^ within the SPM8 software (Welcome Department of Imaging Neuroscience; http://www.fil.ion.ucl.ac.uk/spm), which includes an fMRI rat brain template and the atlas of Paxinos and Watson^40^.

The functional data sets of all individual rats were pre-processed in spmratIHEP with the following major steps. (1) The first 10 volumes of each rat were discarded to allow for magnetization equilibration. (2) Slice timing: the differences in the slice acquisition times were corrected for using slice timing in each rat. (3) Realignment: the temporally processed volumes of each rat were realigned to the first volume to remove head motion, and a mean image was created over the 310 realigned volumes. All rats exhibited less than 1 mm of translation in the *x*, *y*, and *z* axes and 1° of rotation in each axis. (4) Spatial normalization: the realigned volumes were standardized spatially to the Paxinos and Watson space via normalization with the EPI template of a rat brain via their corresponding mean image. Then, all normalized images were resliced to 1.0 × 1.5 × 1.0 mm^3^ voxels. (5) Smoothing: the normalized functional series were smoothed with a Gaussian kernel of 2 × 4 × 2 mm^3^ full width at half-maximum (FWHM).

Using DPARSF (http://rfmri.org/DPARSF), all smoothed images were then band-pass filtered at 0.01–0.08 Hz and further corrected for the effect of head movement by regressing out the translations and rotations of the head that were estimated during image realignment. FC was evaluated using the PAG as the seed region^41^. Finally, to identify differences in FC between the IS group and the Control group, two-sample *T* tests were used. Significant FC was determined based on a voxel-level height threshold of *P* < 0.001 (uncorrected) and a cluster-extent threshold of 20 contiguous voxels.

### Statistical analysis

The SPSS (ver. 20.0; IBM Corp., Armonk, NY, USA) for Windows, GraphPad Prism 5 (GraphPad Software Inc., San Diego, CA, USA) software packages and Adobe Photoshop CS6 (Adobe system Inc., San Diego, CA, USA) were used for the statistical analyses and graph generation, respectively. Levene’s test for homogeneity was used, and non-normally distributed data were analysed using the Kruskal−Wallis test to identify the differences between the groups. Repeated-measures analysis of variance was used to compare the nociceptive behaviors after the data were examined for normality. Least significant difference *T* tests (when the variance was regular) or Dunnett’s T3 tests (when the variance was irregular) were used to compare the differences between the groups. *P* < 0.05 was considered to indicate statistical significance.

## Results

### Behavioral results

Based on previous studies, two types of nociceptive behavior were observed to confirm whether the rats experienced ongoing pain after IS infusion in this study, i.e., face-grooming and quiescent behaviors^29, 34^.

### Mean quiescent behavior over 21 days

**Figure 2:**
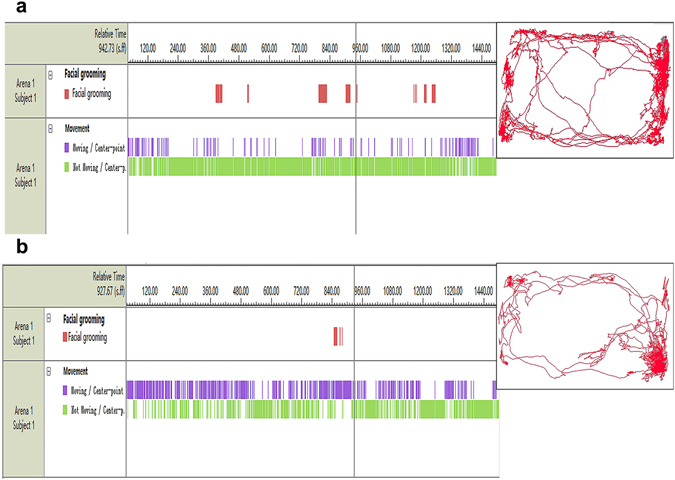
Tracking of rat activity using a video tracking system (EthoVision XT^35^). Rat activity trajectories after infusion. (**a**) An animal’s path after saline infusion. (**b**) After IS infusion, the rats exhibited greater face-grooming and quiescent behavior times. The panels on the left illustrate the time and duration of each behavior. The red bars represent face-grooming activity. The blue bars illustrate moving activity. The green lines show quiescent behavior. The square area represents the open arena, and the representative path of one rat during the 30 min after infusion is shown in red. IS, inflammatory soup.

**Figure 3:**
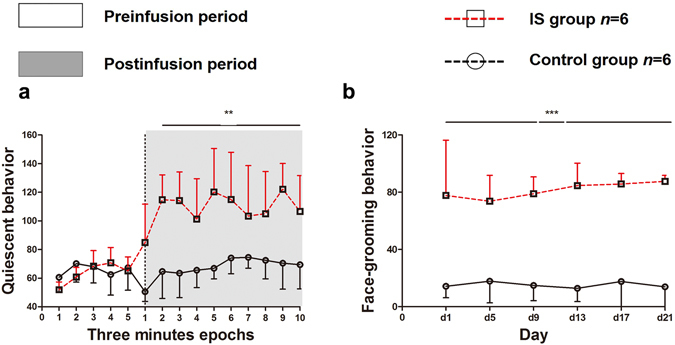
Nociceptive behaviors induced by IS. Average time spent in quiescent behavior (**a**) and face-grooming behavior (**b**) during the 21-day experiment. The mean ± the (SD) values are shown. (**a**) The figure shows the average time (in s) spent in quiescent behavior during the 21 days of the experiment, divided into the preinfusion period (15 min) and postinfusion period (30 min). The shading shows the postinfusion period. Each period was analysed in blocks of 3 minutes and was measured in s. (**b**) The picture shows the average time (in s) spent in face-grooming behavior, which was analysed every 4 days during the 30 min after IS or saline infusion. The IS group showed a significant increase in quiescent behavior compared with the Control group in the postinfusion period and an increase in facial grooming behavior in the day-by-day analysis (**P < 0.01, ***P < 0.001). IS, inflammatory soup.

We analysed the mean ± (SD) of the time that each group displayed quiescent behavior during the 21 days of the experiment. The animal activity trajectories are shown in Fig. 2. The recordings were divided into blocks of 3 minutes; thus, the preinfusion period is illustrated as a five-block period and the postinfusion period is shown as a ten-block period. The results for the quiescent behavior are provided in Fig. 3a. During the preinfusion period, there was no significant difference in quiescent behavior between the groups (*F*_0.05, (3.16)_ = 3.363, *P* = 0.73). However, the time spent in quiescent behavior was greater in the IS group (109 ± 16 s) than the Control group (67 ± 11 s) during the postinfusion period (*F*_0.05, (3.16)_ = 22.398, *P* = 0.001).

### Day-by-day analysis of face-grooming

As illustrated in Fig. 3b, the IS group (81 ± 18 s) exhibited a significant increase in face-grooming behavior over the 21-day period compared with the Control group (5 ± 13 s) in the postinfusion period (*F*_0.05, (3.16)_ = 74.232, *P* < 0.001).

### Functional connectivity analysis

**Figure 4:**
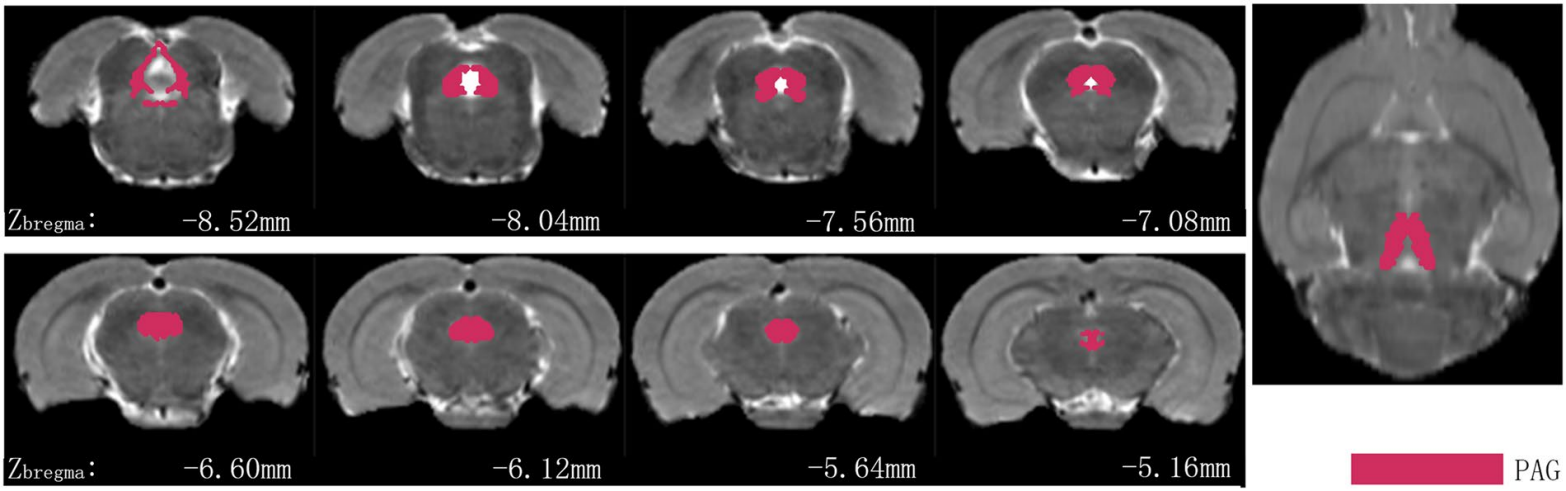
Periaqueductal gray (PAG) seeds across the rats imposed on the T2-weighted MRI template and on the rat atlas structures. The anatomical boundaries for each rat were based on the atlas of Paxinos and Watson^40^. The coronal slices of the PAG are shown in the left two rows, and the right image shows the axial slice of the PAG.

**Figure 5:**
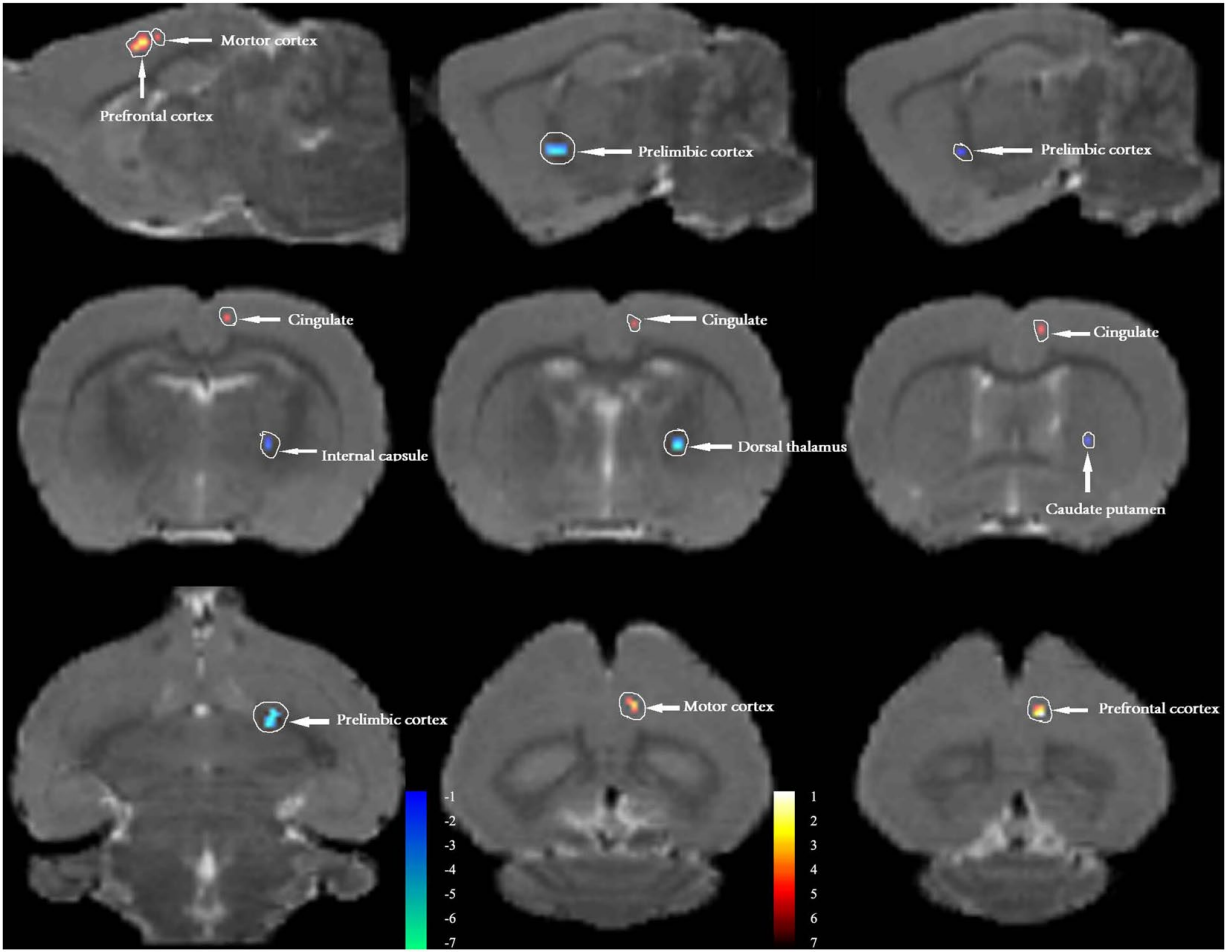
PAG based functional connectivity (FC) in rats induced by dural inflammatory stimulation and in matched controls (day 21). Contrast analysis of the FC differences between the IS group and the Control group revealed significantly increased FC (in red) and decreased FC (in blue) with the PAG in the IS group. Details of the clusters shown are reported in Table. IS, inflammatory soup; PAG, periaqueductal gray.

Anatomical boundaries of PAG seeds are shown in Fig. 4. The rats that received the repeated infusions of IS showed significantly increased functional correlations (*P* < 0.001, uncorrected, extent threshold k = 20 voxels) between the PAG and several cortical regions that are primarily involved in nociception and somatosensory processing compared with the Control rats that received repeated infusions of saline. Areas of note include the prefrontal cortex (i.e., the prefrontal cortex, anterior cingulate cortex, and medial-prefrontal cortex), cingulate gyrus, and motor cortex. The basal ganglia, including the caudate putamen, dorsal thalamus-lateral nucleus group, internal capsule, striatum, and prelimbic cortex, showed relatively decreased FC with the PAG in the rats that received the repeated infusions of IS compared with the Control rats (*P* < 0.001, uncorrected, extent threshold k = 20 voxels). Detailed information is provided in the Table 1 and Fig. 5.

**Table 1:** Brain regions with atypical functional connectivity with the periaqueductal gray in rats induced by dural inflammatory stimulation.

Cluster or region of interest	Coordinates of peak(s) voxel (x, y, z)	Peak *T* value	Effect direction
Cingulate gyrus	0.67, 0.79, −1.08	5.18	IS > Control
Prefrontal cortex (prefrontal cortex, anterior cingulate cortex, medial prefrontal cortex)	0.66, 1.12, 0.12	3.66	IS > Control
Motor cortex	0.80, 0.90, −0.60	7.61	IS > Control
Basal ganglia (caudate putamen, striatum)	2.53, 5.49, −0.12	7.30	Control > IS
Dorsal thalamus, lateral nucleus group	2.28, 5.60, −1.56	4.27	Control > IS
Internal capsule	2.28, 5.55, −1.08	6.54	Control > IS
Prelimbic cortex	−0.35, 2.10, 4.92	4.38	Control > IS

Regions with changes in functional connectivity with the periaqueductal gray were found in rats induced by subcranial (supradural) infusion of inflammatory soup compared with saline-treated control rats. The coordinates according to Paxinos and Watson^40^ are given in mm.

## Discussion

While many preclinical studies based on rats involving pain induced by dural inflammatory stimulation have been performed to elucidate the pathogenesis of migraine^9, 31, 33^, the validity of this model for simulating clinical brain pathophysiology remains unclear. Our study aimed to identify atypical FC of the PAG with other brain regions and reveal brain functional changes that had been induced by dural inflammatory stimulation in male rats. In this study, the EthoVision XT animal-tracking software was used to achieve an objective, accurate and convenient measurement of the time spent in quiescent behavior. Consistent with previous studies^29, 31, 33^, the rats treated with repeated infusion of IS exhibited more extensive quiescent behavior after infusion, which mimics migraineurs who have a tendency to reduce their routine physical activity^5^. No significant differences in quiescent behavior were found between the groups during the preinfusion period, which may indicate that rats in the IS group were in a painless phase. Another pain-related behavior observed in our study was face-grooming. Fifteen face-grooming actions were identified, and these included nine different types of “face wash strokes” of the paw over the face, which were differentiated according to the side (ipsilateral, contralateral, or bilateral) and the facial region to which they were directed^42^. We chose face-grooming behavior as described by Melo-Carillo and Lopez-Avila and Dong *et al*. In contrast to previous findings^29^, longer face-grooming times were found in the IS group in our study. The same result was found in the study performed by Dong *et al*.^34^. The inconsistent results may be caused by different recording times, i.e., 45 min in Melo-Carillo and Lopez-Avila research and 30 min in our study. The most obvious behavioral changes primarily appear before 30 min after IS infusion^29, 31^, and longer recording times may mask the changes. We found increased PAG FC with the prefrontal cortex and cingulate gyrus, and decreased FC of the PAG with the basal ganglia, in the IS group. The increase in PAG FC with the prefrontal cortex and cingulate gyrus is consistent with the rs-FC changes of the PAG in migraine patients^24, 26^. The atypical FC of the PAG in the IS group was associated with repeated stimulation of the meningeal afferents. Thus, rs-FC changes in migraine patients might be a consequence of repeated, long-term nociceptive signalling. Moreover, the results further underscore the utility of repeated IS-infusion in rats as a model of chronic migraine in humans, and the feasibility of FC studies using MRI on rats in future research.

The prefrontal cortex has been suggested to play an important role in controlling functional interactions among areas of the brain related to nociception^43^. Increased FC between the prefrontal cortex and the PAG has been found in chronic back pain^44^ and fibromyalgia^45^, which suggests that dysfunction in the central pain modulation network underlies the chronic pain that these patients experience. Trends toward pain relief by transcutaneous electrical nerve stimulation^46^, distraction in healthy volunteers^47^ and acupuncture in knee osteoarthritis patients^48^ have also been observed, which suggests that pain relief can be achieved by modulating the descending pain modulatory pathway. Compared with healthy subjects, migraineurs have exhibited, in the absence of any pain, elevated FC between the PAG and the prefrontal cortex in previous studies^24, 25^. These data manifested as an impairment of descending pain modulation in interictal migraineurs. The increase in FC between the PAG and the prefrontal cortex in the IS-induced rat model further demonstrates that atypical FC of the PAG with the prefrontal cortex plays an important role in the pathophysiology of pain.

The cingulate cortex has been proposed to play key roles in emotion processing, pain and cognitive control^49, 50^. Atypical FC between the cingulate cortex and the PAG has been demonstrated in pain disorders such as chronic back pain, fibromyalgia, and primary dysmenorrhea^44, 51, 52^. Compared with healthy controls, migraine without aura patients (mean headache frequency, 5.93 per month) is associated with reduced rs-FC between the PAG and the rostral anterior cingulate cortex, which indicates that impairments of the descending pain modulatory system are involved in the neural pathophysiology of migraine^53^. However, research on chronic migraine and migraine with allodynia have revealed increased rs-FC between the PAG and the rostral anterior cingulate cortex, which suggests that atypical rs-FC with affective pain regions may be related to the psychiatric disturbances of chronic migraine and migraine-related allodynia^24, 25^. The inconsistent findings regarding migraine patients may be due to differences in clinical features, such as headache attack frequency, psychiatric disturbances and allodynia. It may be reasonable to conclude that rs-FC between the PAG and the cingulate cortex can be used to monitor disease processes. If the situation can be transformed, the rs-FC between the PAG and the cingulate cortex could also be used for treatment evaluation. In our study, increased FC between the PAG and the cingulate cortex was observed in the IS group, which is consistent with studies performed on chronic migraineurs and migraineurs with allodynia.

The basal ganglia is a major site for adaptive plasticity in the brain, affecting the normal state in a broad range of behaviors^54^ as well as neurological and psychiatric conditions including pain^55, 56^. Several studies have been performed on the basal ganglia for FC analyses in migraine patients^27, 57^. Atypical FC between the basal ganglia and several brain regions within nociceptive and somatosensory processing pathways was observed; and the findings are possibly associated with impaired pain processing and modulatory processes. Again, these findings suggest a significant role of the basal ganglia in the pathophysiology of migraine. Decreased FC between the PAG and the basal ganglia was found in our study, which may support the idea of impairment of the descending pain modulatory pathway in this rat model of recurrent headache. Additionally, the motor cortex FC with the PAG was increased in our study, which may serve a compensatory function, inducing pain relief in this model^58^.

Rs-FC between the PAG and other brain regions in interictal migraineurs has also been found in the anterior insula^24, 26^, amygdala^24, 26^, hypothalamus^25^ and other brainstem regions^25^, as well as in cerebellar regions^25^. Atypical rs-FC between the PAG and cerebral regions such as the anterior insula and amygdala in migraine patients may indicate that pain modulation is more sophisticated in humans than in our rat model of recurrent headache. Previous studies have demonstrated that other brainstem regions such as the dorsal rostral pons^59^ and the TNC^60^, play important roles in the pathogenesis of migraine. Among clinical studies, only one study revealed that migraineurs with severe allodynia have stronger PAG rs-FC to other brainstem regions including the pons and ventral medulla^25^. This study deduced that atypical rs-FC of the PAG to these brainstem regions was associated with migraine-related allodynia. The negative results in other clinical studies and our study may be indicative of consistent functional changes in the brainstem or of brainstem dysfunction resolving in the interictal phase.

The hypothalamus plays critical roles in autonomic and endocrine regulations^61^. The hypothalamus has been implicated in the premonitory symptoms that are frequently experienced by migraineurs such as sleep disorder^62^. The hypothalamic nociceptive modulatory nuclei project to the trigeminocervical complex, and these projections may modulate trigeminovascular nociceptive traffic in migraine patients^8^. A longitudinal functional MRI (fMRI) study on a migraine patient found altered hypothalamic connectivity with the PAG on the day before a headache, and the authors proposed that the real driver of attacks might be the functional changes in hypothalamo–brainstem connectivity^63^. Stress, sleep deprivation, oversleeping and hunger which have close relationships with the hypothalamus, are typical migraine triggers. Whether the dysfunction of the hypothalamus is the driver of migraine attacks or only related to the premonitory symptoms experienced by migraineurs needs further study. There was no atypical FC between the PAG and the hypothalamus in our study, possibly indicating an important difference between our rat model of recurrent headache and changes that precede attacks of migraine.

While there are important benefits of using animal models in brain imaging research, there are several shortcomings in pain models, especially migraine-related pain. Although rats may show nociceptive behavior mimicking the chronic migraine phenotype, whether they are suffering from chronic headache is unknown. Moreover, whereas sub-cortical brain regions are fairly homologous among mammals, the cerebral cortex is much more developed in humans than in rodents. Thus, it is important not to overstate imaging results in rodents. Second, given that pain is a complex experience, consciousness is necessary to experience it^64^. Although several studies have found that the topological features are maintained and that the integrity of the whole brain network can be conserved in the anesthetized brain, these studies were investigating the effects of strong nociceptive stimuli^65^. Our rodent imaging scanning was performed under anesthesia, which may have affected functional activation and filtered out less robust changes. Only spontaneous respiration rate was monitored during the imaging; thus, hypoperfusion artefacts cannot be fully excluded. Finally, only male rats were used in our study; there are some sex differences in pain neurobiology, treatment efficacy, experimental pain responses, and even brain structure^31, 66^. We chose male rats to avoid the possible confounding effects of differences in estrous cycles among the female rats. In addition, the majority of preclinical research has focused on the pain circuitry of male rodents.

## Conclusion

The current study demonstrated atypical FC of the PAG with brain regions primarily involved in nociception, somatosensory processing, emotion processing, and pain modulation in rats following repeated stimulation of meningeal afferents. This result suggests that rs-FC changes in migraine patients may be a consequence of repeated, long-term nociceptive signaling. Similar findings in both the IS-induced model and migraine patients further validate the suitability of the model of recurrent headache induced by repeated infusions of IS, and may provide a new methodological approach for future research using MRI to study migraine.

## References

[1] Stovner L (2007). The global burden of headache: a documentation of headache prevalence and disability worldwide. Cephalalgia: an international journal of headache.

[2] Tepper SJ (2004). Prevalence and diagnosis of migraine in patients consulting their physician with a complaint of headache: data from the Landmark Study. Headache.

[3] Lipton RB, Stewart WF, Diamond S, Diamond ML, Reed M (2001). Prevalence and burden of migraine in the United States: data from the American Migraine Study II. Headache.

[4] Global, regional, and national incidence, prevalence, and years lived with disability for 310 diseases and injuries, 1990-2015: a systematic analysis for the Global Burden of Disease Study 2015. *Lancet (London, England)* **388**, 1545–1602, doi:10.1016/s0140-6736(16)31678-6 (2016). 10.1016/S0140-6736(16)31678-6 PMC5055577 27733282

[5] Headache Classification Committee of the International Headache, S. The International Classification of Headache Disorders, 3rd edition (beta version). *Cephalalgia: an international journal of headache* **33**, 629–808, doi:10.1177/0333102413485658 (2013). 10.1177/0333102413485658 23771276

[6] Natoli JL (2010). Global prevalence of chronic migraine: a systematic review. Cephalalgia: an international journal of headache.

[7] Noseda R, Burstein R (2013). Migraine pathophysiology: anatomy of the trigeminovascular pathway and associated neurological symptoms, cortical spreading depression, sensitization, and modulation of pain. Pain.

[8] Akerman S, Holland PR, Goadsby PJ (2011). Diencephalic and brainstem mechanisms in migraine. Nature reviews. Neuroscience.

[9] Boyer N, Dallel R, Artola A, Monconduit L (2014). General trigeminospinal central sensitization and impaired descending pain inhibitory controls contribute to migraine progression. Pain.

[10] Maizels M, Aurora S, Heinricher M (2012). Beyond neurovascular: migraine as a dysfunctional neurolimbic pain network. Headache.

[11] Behbehani MM (1995). Functional characteristics of the midbrain periaqueductal gray. Progress in neurobiology.

[12] Raskin NH, Hosobuchi Y, Lamb S (1987). Headache may arise from perturbation of brain. Headache.

[13] Gee JR, Chang J, Dublin AB, Vijayan N (2005). The association of brainstem lesions with migraine-like headache: an imaging study of multiple sclerosis. Headache.

[14] Afridi SK (2005). A PET study exploring the laterality of brainstem activation in migraine using glyceryl trinitrate. Brain: a journal of neurology.

[15] Welch KM, Nagesh V, Aurora SK, Gelman N (2001). Periaqueductal gray matter dysfunction in migraine: cause or the burden of illness?. Headache.

[16] Rocca MA (2006). Brain gray matter changes in migraine patients with T2-visible lesions: a 3-T MRI study. Stroke; a journal of cerebral circulation.

[17] Ito K (2016). Detection of changes in the periaqueductal gray matter of patients with episodic migraine using quantitative diffusion kurtosis imaging: preliminary findings. Neuroradiology.

[18] Knight YE, Goadsby PJ (2001). The periaqueductal grey matter modulates trigeminovascular input: a role in migraine?. Neuroscience.

[19] Pozo-Rosich P, Storer RJ, Charbit AR, Goadsby PJ (2015). Periaqueductal gray calcitonin gene-related peptide modulates trigeminovascular neurons. Cephalalgia: an international journal of headache.

[20] Fox MD, Raichle ME (2007). Spontaneous fluctuations in brain activity observed with functional magnetic resonance imaging. Nature reviews. Neuroscience.

[21] Wang K (2007). Altered functional connectivity in early Alzheimer’s disease: a resting-state fMRI study. Human brain mapping.

[22] Zhou Y (2007). Functional dysconnectivity of the dorsolateral prefrontal cortex in first-episode schizophrenia using resting-state fMRI. Neuroscience letters.

[23] Qiu E, Tian L, Wang Y, Ma L, Yu S (2015). Abnormal coactivation of the hypothalamus and salience network in patients with cluster headache. Neurology.

[24] Schwedt TJ (2013). Atypical resting-state functional connectivity of affective pain regions in chronic migraine. Headache.

[25] Schwedt TJ (2014). Allodynia and descending pain modulation in migraine: a resting state functional connectivity analysis. Pain medicine (Malden, Mass.).

[26] Mainero C, Boshyan J, Hadjikhani N (2011). Altered functional magnetic resonance imaging resting-state connectivity in periaqueductal gray networks in migraine. Annals of neurology.

[27] Gao Q (2016). Decreased functional connectivity density in pain-related brain regions of female migraine patients without aura. Brain research.

[28] Wieseler J (2010). A novel method for modeling facial allodynia associated with migraine in awake and freely moving rats. Journal of neuroscience methods.

[29] Melo-Carrillo A, Lopez-Avila A (2013). A chronic animal model of migraine, induced by repeated meningeal nociception, characterized by a behavioral and pharmacological approach. Cephalalgia: an international journal of headache.

[30] Oshinsky ML, Gomonchareonsiri S (2007). Episodic dural stimulation in awake rats: a model for recurrent headache. Headache.

[31] Stucky NL (2011). Sex differences in behavior and expression of CGRP-related genes in a rodent model of chronic migraine. Headache.

[32] Zimmermann M (1983). Ethical guidelines for investigations of experimental pain in conscious animals. Pain.

[33] Su, M. *et al*. Rizatriptan overuse promotes hyperalgesia induced by dural inflammatory stimulation in rats by modulation of the serotonin system. *The European journal of neuroscience*, doi:10.1111/ejn.13296 (2016). 10.1111/ejn.13296 27288111

[34] Dong Z, Jiang L, Wang X, Wang X, Yu S (2011). Nociceptive behaviors were induced by electrical stimulation of the dura mater surrounding the superior sagittal sinus in conscious adult rats and reduced by morphine and rizatriptan benzoate. Brain research.

[35] Noldus LP, Spink AJ, Tegelenbosch RA (2001). EthoVision: a versatile video tracking system for automation of behavioral experiments. Behavior research methods, instruments, & computers: a journal of the Psychonomic Society, Inc.

[36] Tu TW (2016). Radiological-pathological correlation of diffusion tensor and magnetization transfer imaging in a closed head traumatic brain injury model. Annals of neurology.

[37] Tambalo, S. & Peruzzotti-Jametti, L. Functional Magnetic Resonance Imaging of Rats with Experimental Autoimmune Encephalomyelitis Reveals Brain Cortex Remodeling. **35**, 10088-10100, doi:10.1523/jneurosci.0540-15.2015 (2015). 10.1523/JNEUROSCI.0540-15.2015 PMC4495237 26157006

[38] Song T (2015). Functional magnetic resonance imaging reveals abnormal brain connectivity in EGR3 gene transfected rat model of schizophrenia. Biochemical and biophysical research communications.

[39] Nie B (2010). Automatic method for tracing regions of interest in rat brain magnetic resonance imaging studies. Journal of magnetic resonance imaging: JMRI.

[40] Paxinos, G. & Watson, C. The Rat Brain in Stereotaxic Coordinates - The New Coronal Set, Fifth Edition. (2004).

[41] Liang Z, King J, Zhang N (2012). Anticorrelated resting-state functional connectivity in awake rat brain. NeuroImage.

[42] Bart P. Vos, A. M. S. & Raymond J. Maciewicz. Behavioral Evidence of Trigeminal Neuropathic Pain. (1994). 10.1523/JNEUROSCI.14-05-02708.1994 PMC6577477 8182437

[43] Tracey I, Mantyh PW (2007). The cerebral signature for pain perception and its modulation. Neuron.

[44] Yu R (2014). Disrupted functional connectivity of the periaqueductal gray in chronic low back pain. NeuroImage. Clinical.

[45] Truini A (2016). Abnormal resting state functional connectivity of the periaqueductal grey in patients with fibromyalgia. Clinical and experimental rheumatology.

[46] Choi JC (2016). Brain mechanisms of pain relief by transcutaneous electrical nerve stimulation: A functional magnetic resonance imaging study. European journal of pain (London, England).

[47] Valet M (2004). Distraction modulates connectivity of the cingulo-frontal cortex and the midbrain during pain–an fMRI analysis. Pain.

[48] Chen X, Spaeth RB, Retzepi K, Ott D, Kong J (2014). Acupuncture modulates cortical thickness and functional connectivity in knee osteoarthritis patients. Scientific reports.

[49] Etkin A, Egner T, Kalisch R (2011). Emotional processing in anterior cingulate and medial prefrontal cortex. Trends in cognitive sciences.

[50] Shackman AJ (2011). The integration of negative affect, pain and cognitive control in the cingulate cortex. Nature reviews. Neuroscience.

[51] Cifre I (2012). Disrupted functional connectivity of the pain network in fibromyalgia. Psychosomatic medicine.

[52] Wei SY (2016). Changes in functional connectivity of pain modulatory systems in women with primary dysmenorrhea. Pain.

[53] Li Z (2016). Altered periaqueductal gray resting state functional connectivity in migraine and the modulation effect of treatment. Scientific reports.

[54] Graybiel AM (2004). Network-level neuroplasticity in cortico-basal ganglia pathways. Parkinsonism & related disorders.

[55] Obeso JA, Rodriguez MC, DeLong MR (1997). Basal ganglia pathophysiology. A critical review. Advances in neurology.

[56] Chudler EH, Dong WK (1995). The role of the basal ganglia in nociception and pain. Pain.

[57] Yuan K (2013). Altered structure and resting-state functional connectivity of the basal ganglia in migraine patients without aura. The journal of pain: official journal of the American Pain Society.

[58] Peyron R, Faillenot I, Mertens P, Laurent B, Garcia-Larrea L (2007). Motor cortex stimulation in neuropathic pain. Correlations between analgesic effect and hemodynamic changes in the brain. A PET study. NeuroImage.

[59] Afridi SK (2005). A positron emission tomographic study in spontaneous migraine. Archives of neurology.

[60] Wang X, Yu S, Dong Z, Jiang L (2011). The Fos expression in rat brain following electrical stimulation of dura mater surrounding the superior sagittal sinus changed with the pre-treatment of rizatriptan benzoate. Brain research.

[61] Saper CB (2002). The central autonomic nervous system: conscious visceral perception and autonomic pattern generation. Annual review of neuroscience.

[62] Giffin NJ (2003). Premonitory symptoms in migraine: an electronic diary study. Neurology.

[63] Schulte LH, May A (2016). The migraine generator revisited: continuous scanning of the migraine cycle over 30 days and three spontaneous attacks. Brain: a journal of neurology.

[64] Vigil JM, Kruger E (2016). Multi-level selection, social signaling, and the evolution of human suffering gestures: The example of pain behaviors. The Behavioral and brain sciences.

[65] Thompson SJ, Bushnell MC (2012). Rodent functional and anatomical imaging of pain. Neuroscience letters.

[66] Maleki N (2012). Her versus his migraine: multiple sex differences in brain function and structure. Brain: a journal of neurology.

